# Improvement of Temperature Measurement Accuracy of Hot Airflow Using Ultrafine Thermo-Sensitive Fluorescent Wires of Lumisis Phosphor

**DOI:** 10.3390/s24082510

**Published:** 2024-04-14

**Authors:** Shumpei Funatani, Yusaku Tsukamoto

**Affiliations:** Department of Mechanical Engineering, University of Yamanashi, Yamanashi 400-8510, Japan

**Keywords:** LIF (laser-induced fluorescence), two-wavelength LIF, temperature measurement, fluorescent wire, optical sensor, phosphor

## Abstract

In this study, the fluorescence properties of Lumisis, a phosphor that can be easily applied to ultrafine wires, were evaluated. By evaluating the wavelength characteristics of Lumisis phosphor, we investigated the possibility of applying it to a dual-wavelength laser-induced fluorescence (LIF) measurement system and evaluated the accuracy of temperature measurements. The difference between the decrease in the percentage intensities of the red and green fluorescence of Lumisis phosphors showed that two-color LIF was possible. The Lumisis phosphor–mixture ratio was optimized as 1:1.25, and the average measurement error of the fluorescent wire was 0.20 K, as evaluated through uncertainty analysis. Finally, the application of this measurement method to hot air jet phenomena showed that this method accurately captures the temperature changes in hot air, thus proving its validity.

## 1. Introduction

Field measurement techniques for the ambient temperature of gases are in high demand in the industry and are useful for measuring the temperature of spaces such as car air conditioners. The cold and hot air mixing mechanism must be clarified in the design and development of air conditioning equipment and performance evaluations. A well-established method for measuring the airflow temperature distribution is to position several point-measurement temperature sensors, such as thermocouples, and then linearly interpolate the point measurement results. However, the airflow field is disturbed when a large number of temperature sensors are used, and the temperature measurements become inaccurate. Therefore, a line-measurement temperature sensor (in which optical fiber wires are placed in the measurement area) has been proposed. Optical fiber temperature sensors [1,2] are expensive and susceptible to optical fiber damage preventing their widespread use.

In contrast, various flow visualization methods have been developed for the fluid temperature distribution measurement of liquids. To measure hot water in a water tank, a temperature field measurement method that applies the two-color laser-induced fluorescence (LIF) method [3,4,5,6,7] is known. The authors proposed a method for the simultaneous measurement of temperature and velocity fields using a color camera in combination with two-color LIF and particle image velocimetry by optimizing the type and concentration ratio of fluorescent dyes. The measurement system is relatively inexpensive because the optical system is maintenance-free with only one general-purpose red–green–blue (RGB)-type charge-coupled device (CCD) camera used for imaging, and it has high temperature measurement accuracy. Simultaneous temperature and velocity field measurement methods, in which the tracer particles used in the particle image velocimetry (PIV) tracer are temperature-dependent fluorescent particles [8], phosphorescent particles [9,10,11], or temperature-sensitive liquid crystals [12,13,14,15], have also been proposed. However, there are not many reports on the measurement of temperature-sensitive liquid crystals because of their narrow temperature range and the angle dependency of their fluorescence color [13,14].

The two-color LIF method was applied to measure the temperature distribution of the airflow [16]. The authors realized the temperature distribution measurement using the two-color LIF method by spraying fluorescent dye into the atmosphere and irradiating an arbitrary cross-section with a laser light sheet. Propylene glycol was added to the sprayed droplets. The water in the sprayed droplets evaporated, but the propylene glycol did not, and the solution concentrations of propylene glycol and the fluorescent dye as well as the droplet diameter were maintained constant. Droplets containing the fluorescent dye were sprayed into the gas; non-uniformity in the fluorescence intensity was observed, which could be an error factor. In addition, it is impossible to measure the temperature distribution using precision equipment that is vulnerable to water droplets. These problems do not occur when using contact sensors like thermocouples; however, a greater number of measurement points is required for performing 3D measurements. In addition, the internal structure of a thermocouple is a complex four-layer structure consisting of two types of compensation wires, an insulator, and a coating material. Installing a large number of thermocouples, which are not very thin, causes disturbances in the flow field. Surface plasmon resonance temperature sensors using optical fibers [1,2] have complicated optical fiber structures and are expensive to manufacture.

Therefore, the authors proposed a measurement method in which multiple ultrafine fluorescent wires coated with phosphor were placed in the measurement area to determine the temperature distribution in a space without affecting the airflow field.

In a previous study, ultrafine temperature-sensitive fluorescent wires coated with rhodamine B, an organic fluorescent material, were prepared and visualized for measurement [17]. Consequently, we constructed an airflow temperature distribution measurement system based on the temperature dependence of rhodamine B. This ultrafine fluorescent wire has a simple structure and low manufacturing cost because it is a simple fluorescent material applied to an ultrafine wire. However, as the fluorescence intensity of rhodamine B is proportional to the incident light intensity, it was necessary to perform a temperature test before measurement. The disadvantage of this measurement method is that changes in the optical conditions owing to the oscillation of the wire between the temperature test and measurement can cause measurement errors. The two-color LIF method uses rhodamine B and rhodamine 110 and utilizes the difference in the temperature dependence of these two phosphors, and the intensity ratio of the two-color fluorescence can be canceled. However, the fluorescent properties of phosphors are known to deteriorate owing to various factors, and the rate of decrease in fluorescence intensity owing to this deterioration differs for each phosphor, resulting in temperature measurement errors. For example, if an Ar-ion laser beam with an output of approximately 1.0 W (beam cross-section: 9 mm^2^) is continuously injected into a rhodamine B solution (concentration 5.0 × 10^−2^ mg/L) for 50 s, the fluorescence intensity attenuates by approximately 5% [3]. This attenuation depends on the phosphor type. Phosphors used in fluorescent lamps and other applications are materials in which trace amounts of metal ions responsible for luminescence, such as Eu, are added to inorganic ceramic crystals that form the matrix [18]. BAM (BaMgAl_10_O_17_:Eu) is a thermo-sensitive phosphor that can be used in the phosphorescent lifetime method described below owing to its phosphorescent properties and the ability to emit phosphorescent light.

As an inorganic material, this phosphor has excellent heat resistance and can be used for temperature measurements from low temperatures to high temperatures of several hundred degrees Celsius. As they are insoluble in water, alcohols, or organic solvents, they cannot be used for temperature distribution measurements in liquids as a solution of the liquid to be measured. On the other hand, as this phosphor is easily available in particle form with a diameter of a few µm, it can be used as a fluorescent particle with temperature dependence and can therefore be used for the simultaneous measurement of the temperature and velocity.

Therefore, we investigated and developed a method for LIF measurements based on the wavelength distribution of a single type of phosphor and used it to measure the temperature distribution of a transmission oil [19]. Our investigation took advantage of the fact that the peak of the fluorescence wavelength distribution of pyrromethene 597, a type of inorganic phosphor, is temperature-dependent. When performing visualization measurements using a digital SLR camera, the emission wavelength of phosphor should be close to the wavelength range of the RGB image sensor of the digital SLR camera. Hasegawa et al. proposed various europium complexes [20], among which Lumisis phosphors are commercially available [21].

In this study, we selected Lumisis [21], an inexpensive phosphor that can be easily applied to ultrafine wires, and evaluated its fluorescence properties. By evaluating the wavelength characteristics of Lumisis, we examined the possibility of applying it to a two-wavelength LIF measurement system and evaluated the accuracy of the temperature measurements. Finally, we applied this measurement method to hot air jet phenomena.

## 2. Measurement Methods and Experimental Setup

### 2.1. Laser-Induced Fluorescence (LIF)

Laser-induced fluorescence (LIF) is a measurement technique that visualizes the fluorescence of specific molecules using a laser as the light source. If the fluorescence of a fluorophore mixed in a fluid shows temperature- and concentration-dependent brightness and color, then image capture can be used to measure the temperature and concentration distributions. Laser-induced fluorescence (LIF) is a typical, image-based measurement method that is often used to measure water concentration and temperature.

In this study, the two-color LIF method [3] proposed by Sakakibara and Adrian is used to measure the temperature. In this method, two fluorescent dyes with different temperature sensitivities are used as temperature sensors, and the intensity ratio of the emission light from the two fluorescent dyes is used to eliminate the effect of fluctuations in the incident light intensity. The luminescence intensity of the fluorescent dyes is expressed in Equation (1):

(1) $$I=I_{0}C\phi \varepsilon ,$$

where *I* (W/m^3^), the light energy emitted per unit time by a fluorescent dye in a unit microvolume, is proportional to the number of molecules absorbing excitation photons per unit volume per unit time; *I*_0_ (W/m^2^) is the excitation light flux incident on the microvolume; *C* (g/m^3^) is the concentration of the fluorescent dye; ∅ is the quantum yield that indicates the ratio of the absorbed excitation light to the fluorescent emission; and *ε* (m^2^/g) is the absorption coefficient that indicates the ratio of the light intensity absorbed when the excitation light passes through a unit length of the solution of the unit concentration to the incident excitation light intensity. Therefore, the intensity ratio, *I_A_*/*I_B_*, of the fluorescent dyes A and B can be expressed according to Equation (2).

(2) $$\frac{I_{A}}{I_{B}}=\frac{C_{A}\phi_{A}\varepsilon_{A}}{C_{B}\phi_{B}\varepsilon_{B}}.$$

It can be observed from Equation (2) that *I_A_*/*I_B_* is independent of the incident light intensity *I_0_* but depends on the temperature through the ratio of the quantum efficiency ε. Therefore, the temperature can be measured when the following relationships hold:

(1) The relationship between *I_A_*/*I_B_* and the temperature is known.
(2) The concentration ratio *C_A_*/*C_B_* is kept constant.

When measuring the temperature, it is necessary to separate the excitation light and fluorescence using a filter.

### 2.2. Lumisis Phosphor

**Figure 1:**
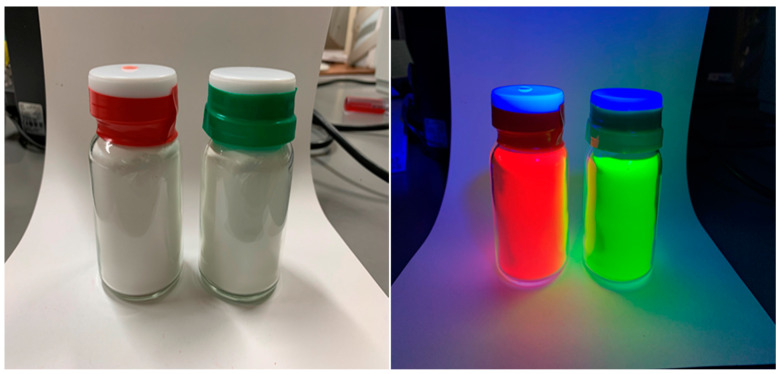
Lumisis phosphors.

Lumisis phosphors, manufactured by Central Techno Co., Ltd., Osaka, Japan [21], are shown in Figure 1. Lumisis Red is composed of 1-Methyl-2-[4-(1,3-dioxoisoindolin-2-yl)-1,3-butadienyl] quinolinium and Lumisis Green is 2-(3-oxoisoindolin-1-ylidene) Methylquinoline. The wavelength of the black light used for emission was approximately 320–380 nm. Lumisis phosphors are white powders that become colorless under sunlight and are transparent when dispersed in resins such as plastic. Lumisis phosphors maintain their strong luminescence even after being exposed to high temperatures of 200 °C for one hour. Because of their toughness against the fluorescence intensity degradation, the accuracy of LIF temperature measurements, in which two types of phosphors are mixed, are not affected. Moreover, the effect of different fluorescence intensities decreases owing to the degradation of the fluorescence intensity of different phosphors. Therefore, we decided to use the Red and Green Lumisis phosphors.

### 2.3. Two-Wavelength Spectral Optics

The following is an imaging system that uses two-wavelength spectroscopic optics. Color changes can be quantified by acquiring the images, visualized with a color CCD or color CMOS camera, and converting them into the RGB or hue–saturation–intensity color space. However, the center wavelengths of the RGB elements of a color camera are approximately 100 nm apart, which limits the ability to measure slight color changes with high precision. In a previous study, we constructed a two-wavelength spectral optical system (center wavelengths: 570 and 610 nm), to improve the accuracy of the temperature measurement in the wavelength range of yellow to red coloration [19]. Pyrromethene 597 phosphor showed a slight temperature dependence on the fluorescence center wavelength [19], allowing for the temperature distribution of the optical fluorescent material in a two-wavelength spectrometer to be measured at two wavelengths. The Lumisis phosphors employed in this study are a combination of green and red phosphors, allowing for two-wavelength measurements using the red and green imaging elements of a color camera without modification.

### 2.4. Measurement of Wavelength Distribution of Lumisis Phosphor

**Figure 2:**
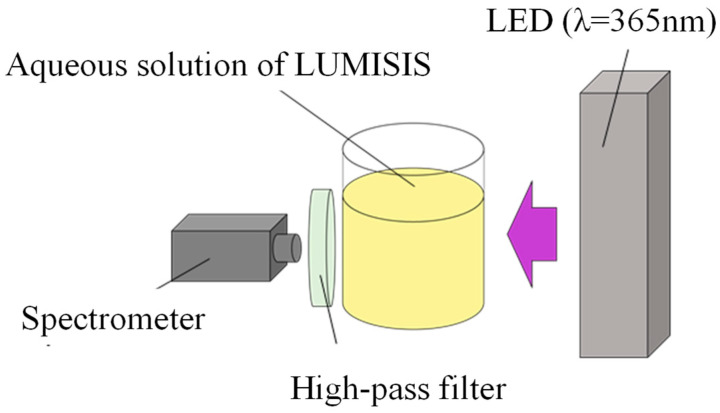
Experimental setup for evaluating temperature dependence.

In this study, we measured the temperature dependence and wavelength distribution of each Lumisis phosphor (Red and Green) to investigate the feasibility of the two-color LIF method. The experimental setup for evaluating the measurement dependence of Lumisis phosphor is shown in Figure 2. The wavelength distribution was measured using a spectrometer (Hopoocolor Co., Ltd., Hangzhou, China, HPCS-300, wavelength: 350–780 nm), where phosphors were dispersed in water because the measurement target needed to be sufficiently large. The aqueous solution was stirred to maintain a constant temperature. A UV LED light (λ = 365 nm) was used as the light source. The aqueous solution was heated to 20–80 °C, and the fluorescence wavelength distribution was measured using a spectrometer at 10 °C intervals. Subsequently, the wavelength distribution was measured. A high-pass filter (transmission wavelength > 470 nm) was used to remove UV light.

### 2.5. Evaluation of Temperature Measurement Accuracy

**Figure 3:**
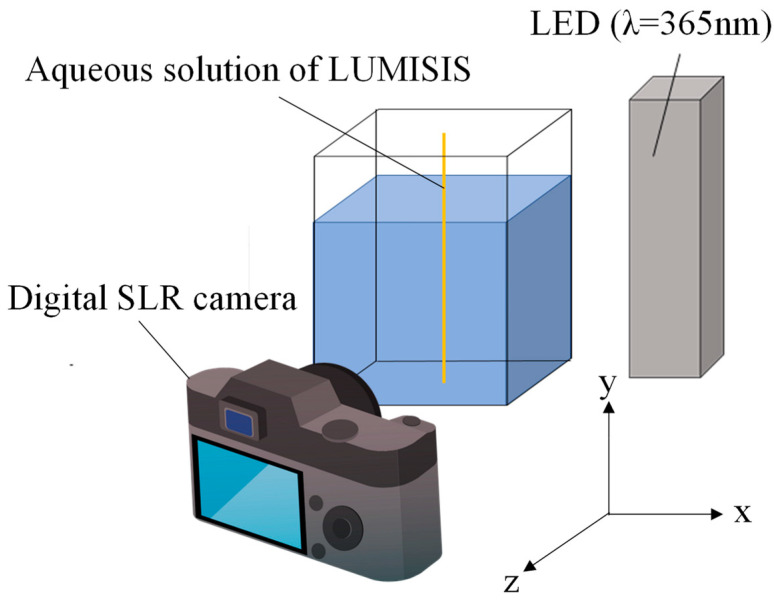
Experimental setup for evaluating temperature measurement accuracy.

The experimental setup for evaluating the measurement error of the Lumisis phosphor wire is shown in Figure 3. The apparatus consisted of a glass tank in which a phosphor wire was submerged. The light source is a UV LED (CCS LDL-100X50UV2-365-ST, λ = 365 nm, beam irradiance: 23 mW/cm^2^) irradiated from the front at an angle. The irradiance was sufficiently small such that the effect of heating on the wire surface was negligible. The visualized images were acquired in the *z*-axis + direction using an ultra-sensitive digital SLR camera (Nikon Corporation, Tokyo, Japan, D7000). The water temperature was raised using a heater and measured at 10 °C intervals from 20 to 50 °C. The water was constantly stirred. By conducting the experiment in water, rather than in air, it was easier to maintain a constant ambient temperature, and the temperature of the entire phosphor wire (in water) could be kept constant. Submerging the wire in water had no effect on the properties of the phosphors because the phosphors were coated with silicone resin. The measurement error was evaluated assuming that the temperature of the entire phosphor wire was constant. A high-pass filter (transmission wavelength > 470 nm) was used to remove UV light. The cut-off frequency of this high-pass filter was lower than the transmission frequency of each RGB image sensor of the color camera; therefore, it was not required. However, it was used to prevent the slight transmission of incident light. This high-pass filter was also used in the experiments described in the subsequent sections.

The following experimental procedure was used to evaluate the measurement error of the Lumisis phosphor wires.

(1) Fill the glass tank with water.
(2) Fix the fluorescent wire vertically at the center of the tank.
(3) Set the camera in an optimal position such that the entire fluorescent wire can be observed.
(4) Shine an ultraviolet LED light on the fluorescent wire.
(5) Increase the water temperature from 20 °C to 50 °C at 10 °C intervals and capture the images of the fluorescent wire at each temperature.
(6) Calculate the temperature of the entire fluorescent wire (at each temperature) from the data obtained via image analysis and compare it with the corresponding temperature in water to evaluate the measurement error.

### 2.6. Experimental Setup for Measurement of Temperature Distribution Using Lumisis Phosphor

**Figure 4:**
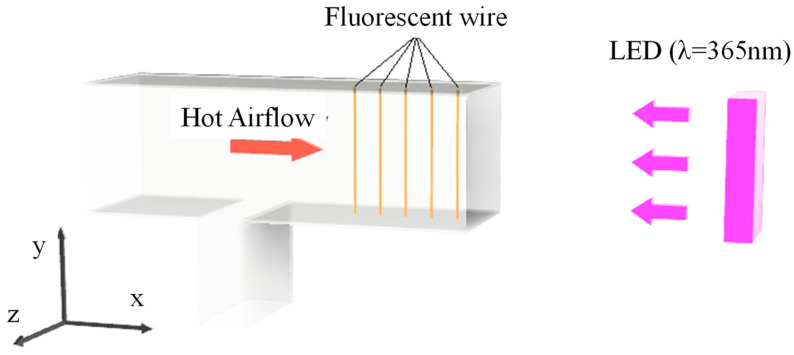
The experimental setup for measuring the temperature distribution using Lumisis phosphor.

Figure 4 shows an evaluation device for a cold and hot air mixing mechanism modeled after a jet of warm air from an automobile air conditioning and heating unit. Hot air was blown from the left side of the device, room-temperature air was blown from below, and the mixed gas was discharged from the right side of the device. For this measurement, several ultrafine stainless steel wires coated with phosphor were placed in the atmosphere. The wires were irradiated with an ultraviolet LED lamp (center wavelength: 385 nm) to obtain the images required for the LIF method. Ultrafine fluorescent wires were placed in the y-direction every Δx = 10 mm, and visualized images were acquired from the *z*-axis + direction using an ultra-sensitive digital SLR camera (Nikon D7000). From these images, the luminance ratio of the R image to the G image (hereafter referred to as the G image) was obtained for the lens, and an equation relating the temperature to the luminance ratio was obtained. The image of the fluorescent wire was taken from a distance of about 0.5 m from the wire. 

The phosphor wire used to measure the temperature distribution was made by spraying a stainless steel wire (0.05 mm in diameter) with fluorescent paint mixed with Lumisis phosphor using an airbrush. The pressure applied to the airbrush was 0.15 Mpa. In this study, fluorescent paint was prepared from a mixture of Lumisis phosphor, thinner, and silicone resin. The resin alternatives for coating the metal wire surfaces were silicone resin and acrylic resin, although acrylic resin has low UV transmittance and interferes with the UV irradiation light hitting the phosphor. Therefore, a two-component silicone resin (Engraving Japan Co., Ltd., Yamanashi, Japan, HTV-4000, viscosity: 7500, elongation: 260%, tear strength: 25 kN/m) was employed. To decrease the viscosity of the silicone resin, it was mixed with a thinner and sprayed over the stainless steel wire. The thinner volatilizes after application. The thickness of the coating was approximately 10 µm. This thickness was visually confirmed using an optical microscope. The luminance of the wire center was obtained, as in a previous study, by calculating the peak value of a quadratic curve approximation of the luminance distribution [17].

**Figure 5:**
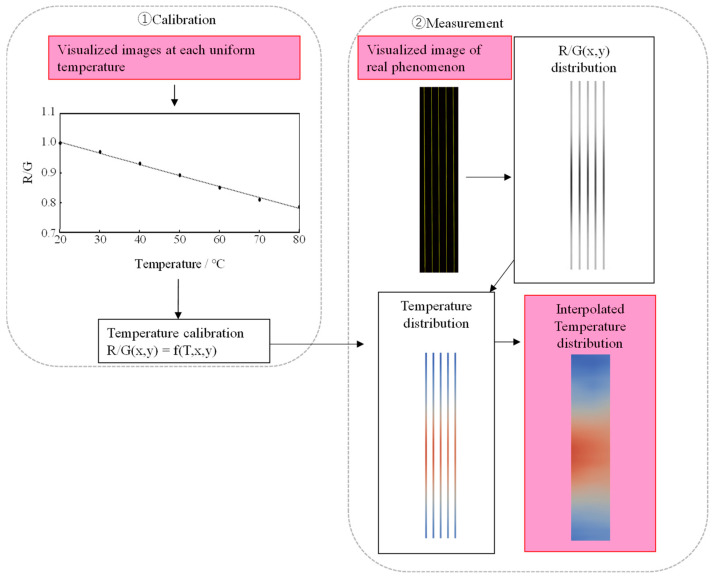
Procedure of temperature evaluation.

The procedure for the temperature measurement (Figure 5) is described below.

(1) Fix the Lumisis phosphor wire in the measurement area of the experimental setup.
(2) Position the camera equipment perpendicular to the measurement area.
(3) Switch off the room light.
(4) Increase the temperature in the measurement area from 20 °C to 80 °C at 10 K intervals using a heater, mix the air to keep the temperature uniform, and then capture images of the fluorescent wire at each temperature.
(5) Calculate the relationship between the temperature and luminance ratio.
(6) Take images and acquire R and G values of the wire surface.
(7) Convert the luminance ratios of the R and G values of the wire surface into temperature values.
(8) Calculate the 2D temperature distribution via the linear interpolation of the obtained temperature values for each wire surface.

## 3. Results

### 3.1. Characteristics of Lumisis Phosphor

**Figure 6:**
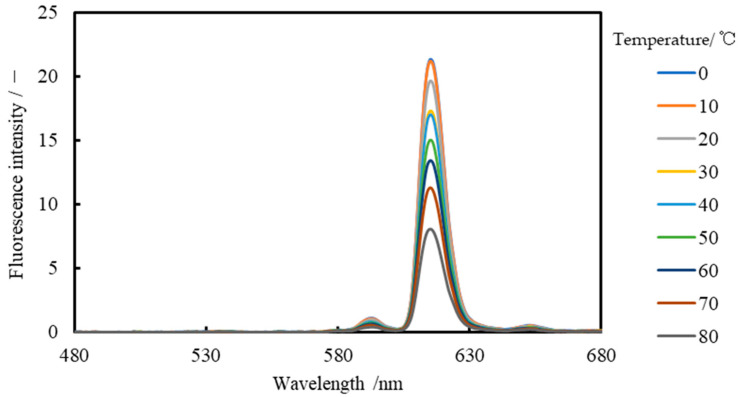
Fluorescence emission spectrum of Lumisis Red (spectrometer).

**Figure 7:**
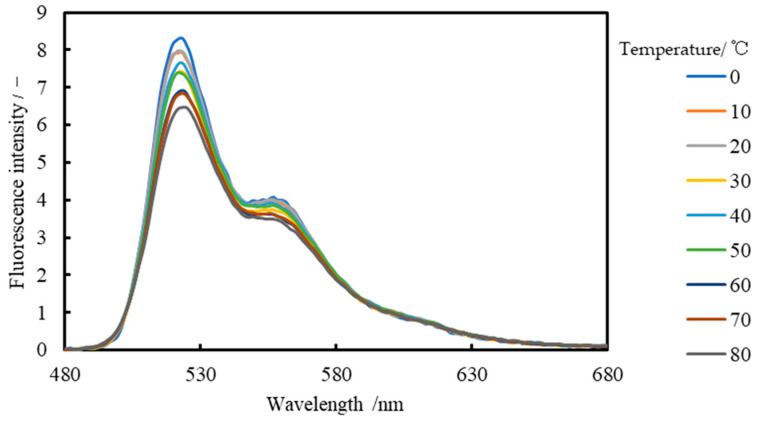
Fluorescence emission spectrum of Lumisis Green (spectrometer).

The wavelength distribution of the Lumisis Red solution is shown in Figure 6 and that of the Lumisis Green solution is shown in Figure 7, respectively. This indicates that the R and G of the Lumisis phosphors are temperature-dependent. The decrease rate of Red is −0.84%/°C and that of Green is −0.30%/°C, indicating that the decrease rate of R is larger than that of G. The peak wavelengths of Red and Green were approximately 615 and 522 nm, respectively, which are approximately 90 nm apart.

### 3.2. Characteristics of Mixed Aqueous Solution of Lumisis Red and Green

**Figure 8:**
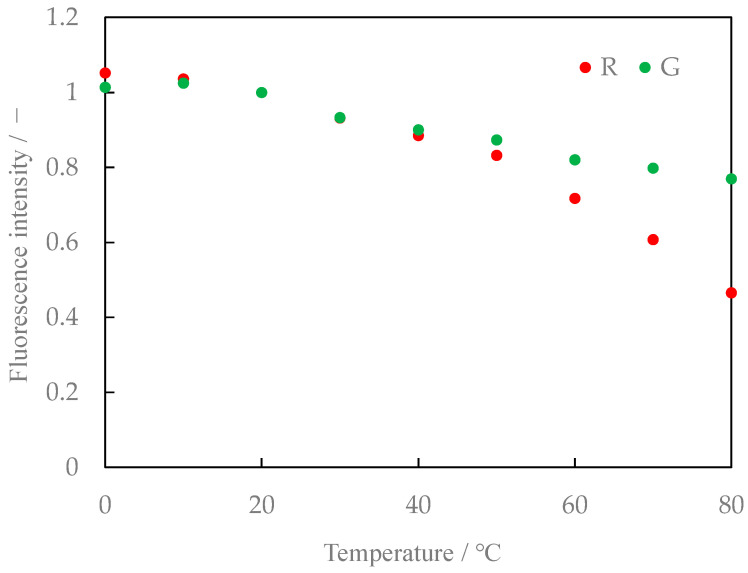
Relationship between temperature and fluorescence intensity (Red/Green = 1:1, digital SLR camera).

**Figure 9:**
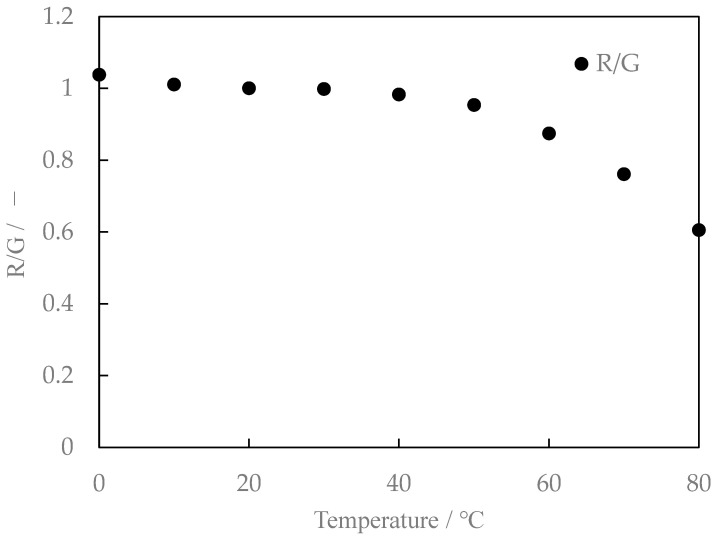
Relationship between temperature and intensity ratio (Red/Green = 1:1, digital SLR camera).

Figure 8 shows the relationship between the temperature and fluorescence intensity of the Lumisis Red and Green mixture at a ratio of 1:1. Figure 9 shows the ratio of the intensities of the Red and Green fluorescence (hereafter referred to as the brightness ratio). The R:G value in Figure 8 and Figure 9 is normalized at 20 °C. The luminance ratio tends to decrease with increasing temperature. This result indicates that two-color LIF measurement is possible using the difference in the ratio of the decrease in the intensity of the Red and Green fluorescence of the Lumisis phosphor.

**Figure 10:**
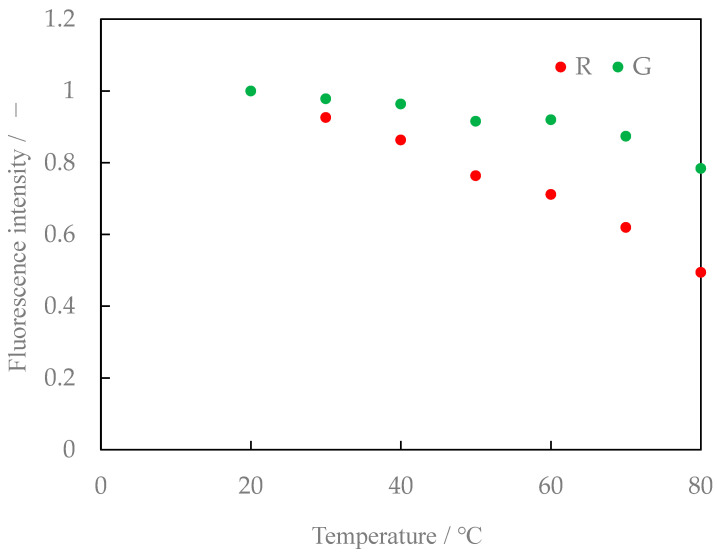
Relationship between temperature and fluorescence intensity (Red/Green = 1:1.25, digital SLR camera).

**Figure 11:**
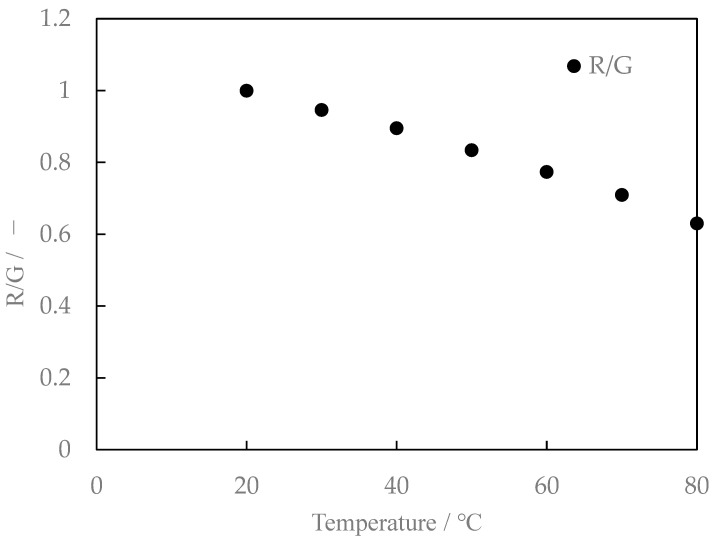
Relationship between temperature and intensity ratio (Red/Green = 1:1.25, digital SLR camera).

We also studied the mixing ratio of the two types of phosphors and found that the temperature sensitivity varied slightly with the mixing ratio. The fluorescence characteristics for Lumisis phosphor with a mixing ratio of R:G = 1:1.25 are shown in Figure 10. The luminance ratios are shown in Figure 11. The R:G value in Figure 10 and Figure 11 is normalized at 20 °C. Comparing Figure 9 and Figure 11 for the temperature dependence of the luminance ratio, it was found that it shows a monotonous decrease in Figure 9 and Figure 11, while Figure 11 (R:G = 1:1.25) shows a constant temperature dependence over a wide temperature range from 20 °C to 80 °C. Based on these results, a mixing ratio of R:G = 1:1.25 for Lumisis phosphors was selected for this study. However, this choice is not final and definitive. The mechanism through which the temperature sensitivity varies with the mixing ratio is unknown but will be clarified in future studies.

### 3.3. Uncertainty Analysis

The total uncertainty in the temperature is defined in Equation (3).

(3) $$T_{c}^{2}=\sum_{i=1}^{N}T_{i}^{2},$$

where *N* is the number of uncertainty factors. Through multiplication, *T_i_* is an uncertainty factor. The uncertainty arises from the thermocouple bias (±0.05 K) and non-uniform temperature field.

To evaluate the accuracy of the temperature detection in the analysis region, the temperature distribution was measured every 10 K in the range of 30–50 °C, and the standard deviations were calculated for the temperature data at all coordinates (Table 1). The results showed that the average accuracy was ±0.09 K (precision error). Integrating the thermocouple errors (±0.05 K) and the visualization errors (Table 1) using Equation (3), the composite uncertainty was estimated to be ±0.10 K (0.20 K in absolute value). This represents an improvement in the average error of ±0.30 K (0.60 K in absolute value) reported in a previous study [16].

## 4. Application to Flow Fields

**Figure 12:**
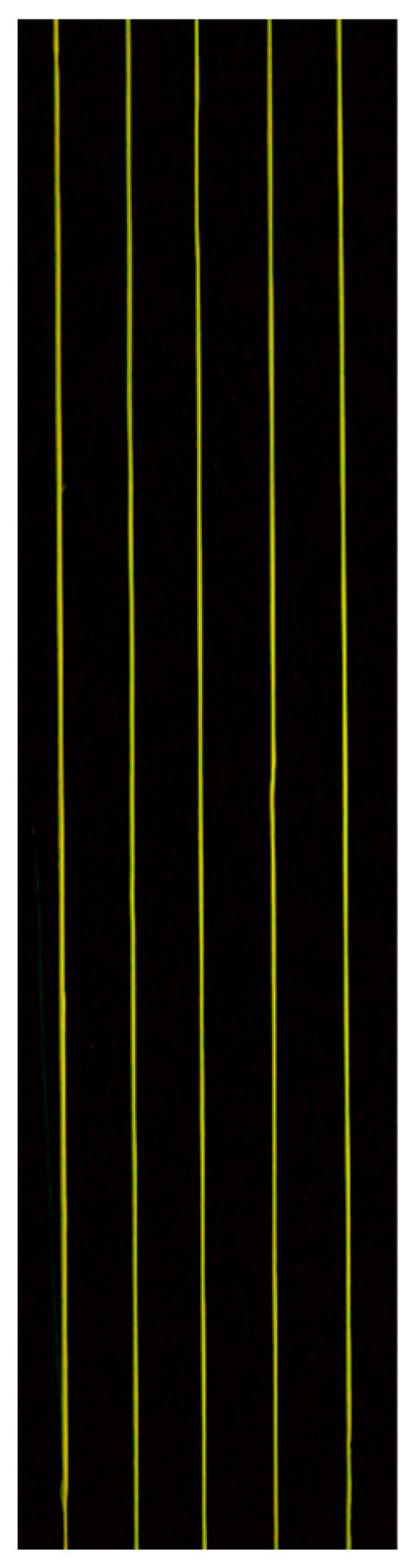
Visualized image of fluorescence wires.

**Figure 13:**
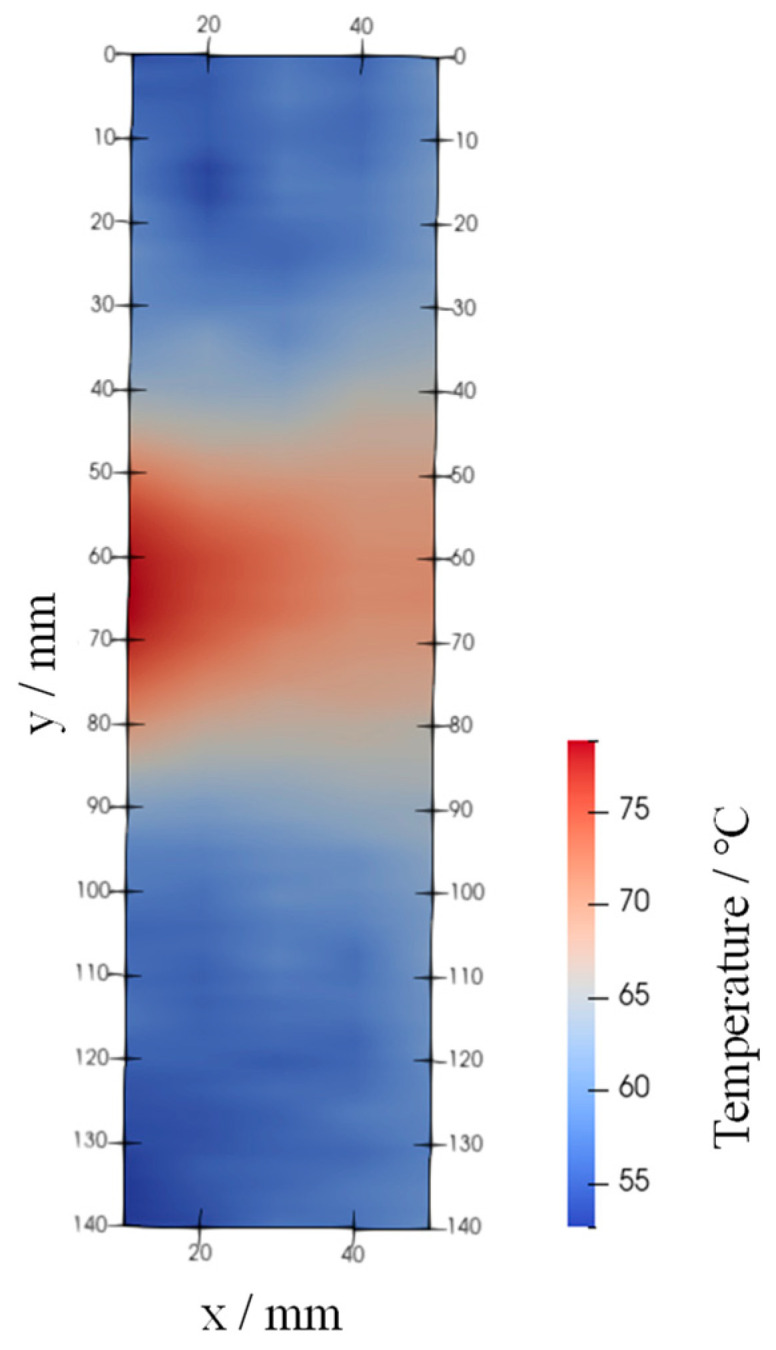
Temperature distribution.

Figure 12 shows an image of the Lumisis phosphor wire (thermocouple temperature = 80 °C) taken using the wind tunnel apparatus shown in Figure 4. The image shows that the wire was green when hot air was blown. This is because the red luminance of the Lumisis phosphor decreases with temperature, and the green luminance increases. Figure 13 shows the 2D temperature distribution in the measurement area. Hot air flowed horizontally from position y = 65 using a heat gun. The thermocouple measured the temperature at (x, y) = (0, 65), and Figure 13 shows that the temperature at the center was higher than the surrounding temperature. The temperature distribution shows the correct trend because the hot air from the heat gun is directed from the center. The temperature change in the hot air was captured from x = 0 to x = 50 because of the improved measurement accuracy in the low-temperature range, and the hot airflow was captured as the hot air rose from y = 65. These results show that the two-color LIF temperature distribution measurement accuracy using Lumisis phosphor was successfully improved.

## 5. Conclusions

In this study, the fluorescence properties of Lumisis phosphors were investigated, and their measurement uncertainties were evaluated. Furthermore, this measurement technique was applied to hot air temperature distribution measurements. The main results obtained in this study are as follows.

(1) The difference in the percentage decrease in the intensity of the Red and Green fluorescence of Lumisis phosphors shows that two-color LIF using Lumisis phosphors is possible.
(2) A Red/Green ratio of 1:1.25 of Lumisis phosphor enabled the highly accurate measurement of temperature changes as well as strong luminescence.
(3) The average measurement error of the fluorescent wire was improved to 0.20 K, compared to the previous studies coating rhodamine B, thereby proving that the temperature measurement accuracy of the proposed method is high.
(4) The results of the application of the proposed measurement method to hot air jet phenomena show that the temperature changes in hot air are accurately captured, thereby proving its validity.

## Figures and Tables

**Table 1:** Temperature measurement error due to visualization of fluorescence wires.

Temperature, °C	30	40	50
Measurement error, K	±0.08	±0.10	±0.08

## Data Availability

Data are contained with the article.
